# Comprehensive Analysis of N^6^-Methyladenosine (m^6^A) Methylation in Neuromyelitis Optica Spectrum Disorders

**DOI:** 10.3389/fgene.2021.735454

**Published:** 2021-11-11

**Authors:** Hong Yang, Yi-Fan Wu, Jie Ding, Wei Liu, De-Sheng Zhu, Xia-Feng Shen, Yang-Tai Guan

**Affiliations:** ^1^ Department of Neurology, The First Rehabilitation Hospital of Shanghai, Tongji University School of Medicine, Shanghai, China; ^2^ Department of Neurology, Renji Hospital, School of Medicine, Shanghai Jiao Tong University, Shanghai, China; ^3^ Department of Neurology, Tongji Hospital, Tongji University School of Medicine, Shanghai, China

**Keywords:** neuromyelitis optica spectrum disorder, immune homeostasis, N^6^-methyladenosine (m^6^A) methylation, differential methylation peaks, MeRIP-seq, UPLC-QQQ-MS

## Abstract

**Background:** N^6^-Methyladenosine (m^6^A) methylation is the most prevalent internal posttranscriptional modification on mammalian mRNA. But its role in neuromyelitis optica spectrum disorders (NMOSD) is not known.

**Aims:** To explore the mechanism of m^6^A in NMOSD patients.

**Methods:** This study assessed the m^6^A methylation levels in blood from two groups: NMOSD patients and healthy controls. Methylated RNA immunoprecipitation Sequencing (MeRIP-seq) and RNA-seq were performed to assess differences in m^6^A methylation between NMOSD patients and healthy controls. Ultra-high performance liquid chromatography coupled with triple quadruple mass spectrometry (UPLC-QQQ-MS) method was performed to check m^6^A level. Differential m^6^A methylation genes were validated by MeRIP-qPCR.

**Results:** Compared with that in the control group, the total m6A level was decreased in the NMOSD group. Genes with upregulated methylation were primarily enriched in processes associated with RNA splicing, mRNA processing, and innate immune response, while genes with downregulated methylation were enriched in processes associated with the regulation of transcription, DNA-templating, and the positive regulation of I-kappa B kinase/NF-kappa B signalling.

**Conclusion:** These findings demonstrate that differential m^6^A methylation may act on functional genes to regulate immune homeostasis in NMOSD.

## Introduction

N^6^-Methyladenosine (m^6^A) is one of the most abundant internal modifications of eukaryotic messenger RNA (mRNA) and plays an important role in gene expression regulatory processes, including the maintenance of stability, splicing and translation (Chen et al., 2019a). Approximately 25% of mRNAs are estimated to contain at least one m^6^A nucleotide site, and most of them occur at the sixth position of adenosine. m^6^A was shown to be specifically catalysed at the consensus gene sequence DRACH (D = A/G/U, R = A/G, H = A/C/U). In addition to m^6^A, other types of posttranscriptional modifications exist, including 5-methylcytosine (m^5^C) and pseudouridine (ψ). To detect regions containing m^6^A peaks, researchers have developed an immunoprecipitation-based method combined with high-throughput sequencing, termed methylated RNA immunoprecipitation sequencing(MeRIP-seq) (Dominissini et al., 2012). To determine m^6^A level, researchers have developed the UPLC-QQQ-MS method, which has the advantages of short analysis time, good resolution and high sensitivity (Su et al., 2014).

m^6^A modification is dynamic and reversible and regulated by m^6^A methyltransferases (writers), demethylases (erasers) and reading proteins (readers). Writers include Methyltransferase Like 3 (METTL3), methyltransferase-like 14 (METTL4) and Wilms tumour 1-associated protein (WTAP). METTL3 catalyses the production of m6A; METTL4 forms a complex with METTL3, participates in interactions with target mRNA and recruits RNA, and WTAP stabilizes the complex (Liu et al., 2014; Ping et al., 2014; Lin et al., 2016). Erasers, including fat and obesity-associated protein (FTO) and alk B homologue 5 (ALKBH5), catalyse the demethylation of bases that have been modified by m^6^A (Jia et al., 2011; Zheng et al., 2013). Readers, including YTH N6 methyladenosine RNA-binding protein 1–3 (YTHDF1-3) and insulin-like growth factor 2 mRNA-binding protein 1–3 (IGF2BP1-3), are also necessary for this process (Wang et al., 2014; Wang et al., 2015; Hanniford et al., 2020). These proteins recognize sites of mRNA methylation and affect RNA metabolism directly or indirectly. Overall, m6A-modifying enzymes play important roles in tumorigenesis and in the development of the human central nervous system (Lin et al., 2017; Zhang et al., 2018; Heck et al., 2020; Xue et al., 2021). In recent years, studies have revealed that m^6^A participates in regulating the immune microenvironment (Chen et al., 2019b; Tang et al., 2020; Xu et al., 2020), but its involvement in NMOSD is unknown.

NMOSD is a kind of recurrent antibody-mediated neuroimmune disease, in which the optic nerve, spinal cord, and area postrema of the medulla are damaged, often causing severe symptoms (Wingerchuk et al., 2015). The specific diagnostic and pathogenic biomarker for NMOSD is aquaporin 4-immunoglobulin IgG (AQP4-IgG), which binds to the foot processes of astrocytes, activating complement and thereby recruiting neutrophils and eosinophils; this leads to astrocyte death, followed by axonal degeneration and oligodendrocyte injury (Lennon et al., 2004). The prevalence and incidence of NMOSD are 0.52–4.4 and 0.05–0.40 per 100,000, respectively (Pandit et al., 2015). Approximately 60% of NMOSD patients relapse within 1 year and approximately 90% within 5 years. Furthermore, approximately 50% of NMOSD patients require a wheelchair within 5 years of their diagnosis due to limb paralysis, and 62% become blind, affecting their quality of life and imposing a heavy burden on patients, families and society (Pittock and Lucchinetti, 2016). Currently, methylprednisolone and plasma exchange used in the acute stage as well as rituximab, mycophenolate mofetil and azathioprine used in the remission stage are only partially effective and do not substantially reduce the high disability associated with the disease or its recurrence. Therefore, it is necessary to further understand the pathogenesis of NMOSDs and explore more effective drugs.

Regardless, the m^6^A status has not been assessed in NMOSD. To investigate differences in m^6^A modification patterns between healthy controls (HCs) and NMOSD patients, we performed UPLC-QQQ-MS to assess the m^6^A level and MeRIP-seq to identify the first known transcriptome-wide m^6^A sites in NMOSD patients. In general, the total m6A level was decreased in patients compared to HCs. We also detected 3,371 differentially methylated peaks within mRNAs and 95 within long noncoding RNAs (lncRNCs, *p* < 0.05). Intriguingly, mRNAs harbouring differentially methylated peaks were shown to be involved in many important biological pathways associated with immune homeostasis.

## Materials and Methods

### Blood Collection From NMOSD Patients and HCs

(1.1) Inclusion criteria: 1) aged 18–75 years old; 2) meeting the diagnostic criteria for NMOSD (Wingerchuk et al., 2015); 3) within 30 days of onset in the acute phase and not receiving relevant drug treatment; and 4) provision of signed informed consent.
(1.2) Exclusion criteria: 1) severe mental symptoms precluding cooperation with treatment; 2) no informed consent signed; 3) receiving immunosuppressive therapy within 30 days of new clinical symptoms; 4) other autoimmune diseases or tumours; 5) new clinical symptoms and not receiving relevant treatment but with clinical symptoms for more than 30 days before seeing a doctor.
(1.3) On the second day after admission, 10 ml of blood was collected into a BD Paxgene blood RNA tube; the samples were stored at −4°C for 24 h and then at −80°C for preparation of RNA.
(1.4) In total, six healthy individuals with no acute or chronic illness from the Health Care Centre of the First Rehabilitation Hospital of Shanghai were enrolled in the study as a control group.

### RNA Preparation and Quality Control

Total RNA was extracted from whole blood stored in Paxgene blood RNA tubes by using a Paxgene Blood RNA Extraction Kit (PreAnalytiX, Qiagen). A Qubit fluorometer was used to quantify RNA at 260/280 nm, and an Agilent 2,100 Bioanalyzer was employed to determine the RNA integrity number (RIN) and 28S/18S values for quality control (RIN ≥ 6.0 and 28S/18S ≥ 0.7) and confirmed by electrophoresis on a denaturing agarose gel.

### UPLC-QQQ-MS Method

Analysis of m6A level was performed on the UPLC-QQQ-MS system, consisting of a Waters Acquity UPLC (Waters Corporation, Massachusetts, United States) and an AB SCIEX 5500 QQQ-MS instrument (AB Sciex LLC, Framingham, United States). As previously described (Su et al., 2014), we prepared a mixture containing all of the cofactors and enzymes needed for hydrolysis of the RNA into nucleosides. Then, 5 µl of the mixture was added to each sample of RNA, and the final volume was brought to 25 µl with RNase-free water. The samples were incubated at 37°C for 4 h, after which 75 µL of methanol was added to the system. Different dilutions were used for detection. UPLC separation was performed on an Acquity UPLC BEH amide column (1.7 µm, 2.1 mm × 100 mm, Waters Corporation, Massachusetts, United States) with a flow rate of 0.35 ml/min at 40°C. Formic acid in water (0.1%, v/v, solvent A, Aladdin, Shanghai) and methyl cyanides (v/v, solvent B, Sigma-Aldrich, Shanghai) were employed as the mobile phase. Mass spectrometry detection was performed in positive electrospray ionization mode, and multiple reaction monitoring (MRM) was used to monitor target nucleosides by using the following mass transitions: (precursor ions → product ions) of m^6^A (282.1 → 150.1), A (268 → 136), U (245 → 113), G (284 → 152), C (244 → 112), and I (269 →137). Quantification was performed using a standard curve originating from A and m^6^A. The calculated m^6^A/A ratio was used to indicate the m^6^A level. To achieve maximal detection sensitivity, we optimized the MRM parameters of all nucleosides.

### RNA MeRIP-Seq Library Construction and Sequencing

MeRIP-seq was performed by Biotechnology Corporation (Shanghai, China). As previously described (Dominissini et al., 2012), GenSeq^®^ m6A MeRIP KIT (GenSeq, China) was performed, and the protocol involved the following four steps. Step 1: RNA fragmentation. The RNA was randomly divided into approximately 200-nt segments by using fragmentation buffer and stop buffer. The sizes and concentrations of the RNA fragments were detected by an Agilent Bioanalyzer and an Agilent RNA 6000 Pico kit (Bioptic Inc., Taiwan, China). Three micrograms of fragmented RNA was used as the input group and stored at −80°C. The remaining fragmented RNA was used for subsequent immunoprecipitation experiments. Step 2: Preparation of immunoprecipitated magnetic beads. The porcine gastric mucine (PGM) magnetic beads were gently blown with a pipette to promote full suspension, after which they were washed with 1 × IP buffer, and an m^6^A antibody was added. Step 3: Immunoprecipitation. The MeRIP reaction solution, which included 50 µl of fragmented RNA, 150 µl of nuclease-free water, and 50 µl of 5 × IP buffer, was prepared. Next, 250 μL of the reaction solution was added to the magnetic beads prepared in step 2. The magnetic beads were washed with 1 × IP buffer, LB buffer, and HS buffer separately and repeatedly. Step 4: RNA purification. The magnetic beads were resuspended in RLT buffer, washed with 75% ethanol, and then completely resuspended in 11.25 µl of nuclease-free water. The eluted RNA was transferred to a new centrifuge tube and then immediately used for subsequent experiments or stored at −80°C. Both the input samples without immunoprecipitation and the m^6^A IP samples were used for RNA-seq library generation. The library quality was evaluated with a Bioptic Qsep100 Analyser (Bioptic Inc., Taiwan, China). Library sequencing was performed on an Illumina NovaSeq 6,000 (LC-Bio Technology) instrument with 150 bp paired-end reads.

FastQC was applied to analyse the quality of the sequencing data by assessing the sequencing quality distribution, base content distribution, and proportion of repeated sequencing fragments. HISAT2 software was used to compare the filtered clean reads with the human reference genome (GRCh38/hg38) and thereby obtain unique mapped reads for further analysis. Peak calling was carried out with exomePeak software. After obtaining the peak, the gene structure locations of the peaks and the overall distribution characteristics were determined, and peak annotation analysis was performed with HOMER software (http://homer.ucsd.edu/homer/ngs/peakMotifs.html).GO analysis is used to assess the function of genes from various aspects and is divided into three main categories: biological process (BP), molecular function (MF) and cellular component (CC). The mRNAs of genes modified by m^6^A were evaluated based on GO annotations in the BP, MF, and CC categories in the database, and Fisher’s test was used to determine the significance level (*p*-value) of each category to screen for significant GO terms. KEGG analysis was based on the gene annotations, and selected genes with mRNA m^6^A modification were annotated according to their associated KEGG pathways. The significance level (*p*-value) was determined by Fisher’s test to screen significant pathway terms related to m^6^A gene enrichment.

### Methylated RNA ImmunoprecipitationeRIP-qPCR

MeRIP-qPCR was performed by ChoudSeq Biotech Inc. (Shanghai, China). Its analysis was used according to a previously reported method (Dominissini et al., 2012), as shown in the RNA MeRIP-seq library construction and sequencing section. m6A enrichment was determined by qPCR analysis. Reverse transcription was performed using a SuperScriptTM III Reverse Transcriptase kit (Invitrogen) for mRNA. For relative qRT-PCR, qPCR SYBR Green master mix (CloudSeq)was used to generate mRNA cDNA according to the manufacturer’s instructions. The reactions were performed on a QuantStudio five Real-Time PCR System (Thermo Fisher). The expression levels of mRNAs normalized to those of the endogenous control were calculated using the 2^−ΔΔCT^ method and are presented as the fold change relative to the control group. % (IP/Input) was used to calculate the differences.The sequences of the primers utilized in this study are listed in Additional file 1: Supplementary Table S1.

### Data Analysis

Data are presented as the mean ± SD. Significance differences between groups were determined by Student’s t-test using GraphPad Prism six sofeware. Significance was established at *p* < 0.05.

## Results

### Demographic and Clinical Features of the Patients With NMOSD

We enrolled three NMOSD patients and three HCs for MeRIP-seq, with mean ages of 47.33 ± 2.082 and 47.33 ± 3.055, respectively (*p* > 0.05). The female: male ratio was 3:0 in both groups (*p* > 0.05). The current disease duration at the time of recruitment was 5 ± 2 days (Table 1). We enrolled another three NMOSD patients and three HCs for MeRIP-qPCR validation, with mean ages of 59.00 ± 6.083 and 59.67 ± 6.807, respectively (*p* > 0.05). The female:male ratio was 3:0 in both groups (*p* > 0.05). The current disease duration at the time of recruitment was 5 ± 2 days (Table 1).

**TABLE 1:** Characteristics of subjects and m^6^A levels in NMOSD patients and healthy controls.

Item	NMOSD (n = 3) for MeRIP-seq	HC (n = 3) for MeRIP-seq	*p*-value	NMOSD (n = 3) for MeRIP-qPCR	HC(n = 3) for MeRIP-qPCR	*p*-value
Mean age (mean ± SD; years)	47.33 ± 2.082	47.33 ± 3.055	*p* > 0.05	59.00 ± 6.083	59.67 ± 6.087	*p > 0.05*
Sex (female/male, %)	3/0 (100,0)	3/0 (100,0)	*p* > 0.05	3/0 (100,0)	3/0 (100,0)	*p > 0.05*
Anti-AQP4 antibody (positive/negative, n,%)	3/0 (100,0)			3/0 (100,0)		
Current disease duration (mean ± SD; days)	5 ± 2			5 ± 2		
m^6^A level (mean ± SD; %)	0.09531 ± 0.009259	0.1399 ± 0.02533	*p* < 0.05	*-*	*-*	*-*

Note:SD,standard deviation; Anti-AQP4, antibody,anti-aquaporin-4 antibody.

### Levels of m^6^A in NMOSD Patients and HCs

**FIGURE 1:**
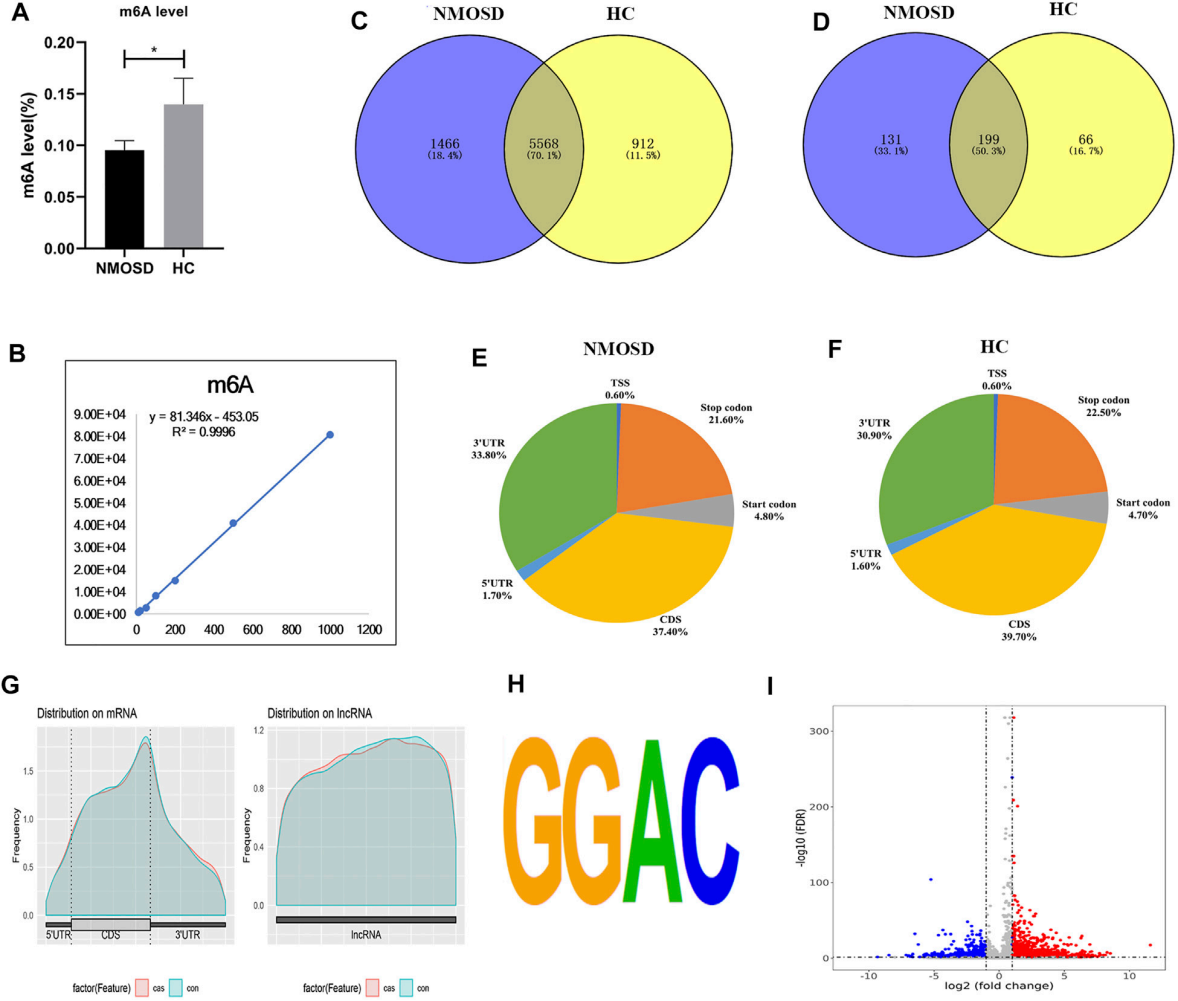
Overview of N^6^-methyladenosine methylation in NMOSD patients and HCs. **(A)** m^6^A levels in NMOSD and HCs,*p < 0.05,Student’s *t*-test. **(B)** *R* ^2^ was greater than 0.99, indicating that the linear relationship of the standard curve is good. **(C)** Venn diagram showing the overlap of m^6^A peaks with mRNA in NMOSD patients and HCs. **(D)** We used a Venn diagram to depict the overlap of m^6^A peaks with lncRNAs in NMOSD and HCs. **(E)** We used pie charts to show the accumulation of m^6^A peaks along transcripts in NMOSD. **(F)** We used pie charts to display the accumulation of m^6^A peaks along transcripts in HCs. **(G)** Metagene plot of peak distribution in RNA structures (NMOSD and HCs). **(H)** The consensus motif for m^6^A modification is “GGAC”.**(I)** Volcano map of differential m^6^A modification. Diff.log2 FC < 0 indicates hypomethylation, indicating that the site was demethylated under treatment conditions; Diff.log2 FC > 0 indicates hypermethylation, indicating hypermethylation of this site under treatment conditions. Diff.lg.fdr: log10 (FDR) of differential methylation analysis is the log10 conversion value of the FDR value obtained from the differential analysis. Red indicates hypermethylation, and blue indicates demethylation. Red and blue represent genes with a more than 2-fold difference in m^6^A modification.

The levels of m^6^A differed significantly between the NMOSD patients and HCs (0.09531 ± 0.009259 (%), 0.1399 ± 0.02533 (%); *p* < 0.05) (Table 1; Figure 1A). Additionally, *R* ^2^ was greater than 0.99, indicating that the linear relationship of the standard curve is good (Figure 1B).

### Sequencing Statistics and Quality Control

Some joint and low-quality sequences were abtained in the original data, and adaptor and low-quality data were removed to abtain clean reads. In the MeRIP-seq library, 6,308,338,650, 7,779,927,600 and 6,841,024,050 total bases and 6,125,183,549, 7,628,452,162 and 6,635,546,539 valid total bases were abtained, and the effective bases accounted for 97.10, 98.10 and 97.00% of the totals, respectively. In the HC blood samples, 6,495,933,300, 6,730,312,650 and 6,736,672,200 total bases and 6,292,021,712, 6,453,732,389 and 6,604,071,380 valid total bases were obtained, with the effective bases accounting for 96.90, 95.90 and 98.00% of the totals, respectively. In the RNA-seq library, the effective bases accounted for more than 90% of the total bases in both the NMOSD and HC groups. The results are shown in Table 2.

**TABLE 2:** Summary of reads quality control analysis.

SampleName	TotalReads_Before	Totalbase_Before	Totalbase_After	BaseFilter% (%)
Patient1-IP	42,055,591	6,308,338,650bp	6,125,183,549bp	97.10
Patient2-IP	51,866,184	7,779,927,600bp	7,628,452,162bp	98.10
Patient3-IP	45,606,827	6,841,024,050bp	6,635,546,539bp	97.00
Control1-IP	43,306,222	6,495,933,300bp	6,292,021,712bp	96.90
Control2-IP	44,868,751	6,730,312,650bp	6,453,732,389bp	95.90
Control3-IP	44,911,148	6,736,672,200bp	6,604,071,380bp	98.00
Patient1-Input	44,180,024	6,627,003,600bp	6,300,789,080bp	95.10
Patient2-Input	57,064,051	8,559,607,650bp	8,145,517,911bp	95.20
Patient3-Input	39,713,253	5,956,987,950bp	5,475,127,085bp	91.90
Control1-Input	43,211,484	6,481,722,600bp	6,080,268,117bp	93.80
Control2-Input	43,081,347	6,462,202,050bp	6,047,838,238bp	93.60
Control3-Input	40,372,667	6,055,900,050bp	5,664,859,110bp	93.50

### Mapping Reads to the Reference Genome

We used HISAT2 to map reads to the GRCh38/hg38 genome with default parameters. Detailed statistical analyses were performed by comparing reads with reference sequences. In the MeRIP-seq library, the mapping ratios were 94.37, 87.19 and 91.55% in NMOSD patients, and 92.64, 93.87, 89.08% in HCs. In the RNA-seq library, the mapping ratios were 91.07, 89.26 and 86.16% in NMOSD patients and 89.31, 90.42, 88.04% in HCs. The ratios of unique mapped reads are shown in Table 3.

**TABLE 3:** Summary of reads mapped to the GRCh38/hg38 reference genome.

Item	All	UnMapped	UniqueMapped	UniqueMappedRate (%)	RepeatMapped	Mapped	MappedRate (%)
Patient1-IP	42,055,591	2,367,986	38,154,965	90.73	1,532,640	39,687,605	94.37
Patient2-IP	51,866,184	6,643,849	41,866,190	80.72	3,356,145	45,222,335	87.19
Patient3-IP	45,606,827	3,854,600	39,952,635	87.60	1,799,592	41,752,227	91.55
Control1-IP	43,306,222	3,188,802	38,005,282	87.76	2,112,138	40,117,420	92.64
Control2-IP	44,868,751	2,751,102	40,569,248	90.42	1,548,401	42,117,649	93.87
Control3-IP	44,911,148	4,905,279	36,597,922	81.49	3,407,947	40,005,869	89.08
Patient1-Input	44,180,024	3,943,392	36,724,619	83.12	3,512,013	40,236,632	91.07
Patient2-Input	57,064,051	6,131,507	40,183,337	70.42	10,749,207	50,932,544	89.26
Patient3-Input	39,713,253	5,497,001	31,232,336	78.64	2,983,916	34,216,252	86.16
Control1-Input	43,211,484	4,620,990	34,806,849	80.55	3,783,645	38,590,494	89.31
Control2-Input	43,081,347	4,127,518	35,559,483	82.54	3,394,346	38,953,829	90.42
Control3-Input	40,372,667	4,830,090	31,472,902	77.96	4,069,675	35,542,577	88.04

### General Features of m^6^A Methylation in NMOSD Patients and HCs

MeRIP-seq analysis of RNA derived from whole blood revealed 14,444 nonoverlapping m^6^A peaks within 7,034 coding transcripts (mRNAs) and 440 nonoverlapping m^6^A peaks within 330 lncRNAs in the NMOSD group; there were three biological replicates. In the HC group, there were 12,806 nonoverlapping m^6^A peaks within 6,480 coding transcripts (mRNAs) and 369 nonoverlapping m^6^A peaks within 265 lncRNAs, with three biological replicates. Of these, 5,568 coding transcripts (70.1%) within mRNAs (Figure 1C) and 199 long noncoding transcripts (50.3%) within lncRNAs (Figure 1D) overlapped between the NMOSD patients and HCs. Of these, there were 3,600 differential peaks (*p* < 0.05), including 3,371 differentially methylated peaks within mRNAs and 95 within lncRNAs (*p* < 0.05) (Figure 1I).

To analyse the distribution profiles of m^6^A peaks within mRNAs, the peaks were categorized into five transcript segments: the 5’UTR; start codon (400 nucleotides centred on the start codon); coding sequence (CDS); stop codon (400 nucleotides centred on the stop codon); and 3’UTR. In our study, m^6^A was most often detected in the CDS, with some sites being observed near the 3’UTR and stop codon (Figures 1E–G).

Motif analysis of peaks within mRNAs with the highest scores (*p*-value = 1e-141) obtained from three biological replicates revealed a consensus sequence (GGAC) as well as other motifs in the NMOSD and HC samples (Figure 1H), indicating the reliability of the data.

### Distribution of Differentially Methylated m^6^A Sites

In total, we identified 3,371 differentially methylated m^6^A sites (DMMSs) within mRNAs, of which 68.5% (2,310*/*3,371) showed significantly increased methylation and 31.5% (1,061/3,371) significantly reduced methylation (NMOSD vs. HCs; Table 4). We also identified 95 DMMSs for lncRNAs, of which 68.5% (71*/*95) exhibited significant increased methylation levels and 31.5% (24/95) exhibited significantly decreased methylation levels (Table 4). Tables 5, 6 provide the top ten m^6^A sites displaying increased and reduced methylation levels with the highest fold change values (Tables 5, 6).

**TABLE 4:** General numbers of differentially methylated peaks and genes.

Item	Increased methylation peak	Increased methylation gene	Decreased methylation peak	Increased methylation gene
mRNA	2,310	1,855	1,061	974
lncRNA	71	63	24	24

**TABLE 5:** Top ten increased methylation peaks.

Chromosome	thickStart	thickEnd	Gene name	Fold change	Class
14	74,736,350	74,736,560	FCF1	11.6	three_prime_utr
17	62,551,671	6,2,551,852	AC008026.3	8.53	exon
12	6,593,496	6,594,518	CHD4	8.28	CDS
12	68,670,970	68,671,151	RAP1B	8.15	three_prime_utr
1	16,574,534	16,574,684	NBPF1	8.02	CDS
17	28,042,829	28,043,009	NLK	7.99	CDS
1	211,574,019	211,574,200	SLC30A1	7.75	three_prime_utr
1	1,340,169	1,341,701	DVL1	7.75	CDS
6	33,663,772	33,665,055	ITPR3	7.7	CDS
15	34,980,730	34,980,881	ZNF770	7.67	three_prime_utr

Note: thickStart/thickEnd: start/end position of the differentially methylated RNA peak; CDS, coding sequence.

**TABLE 6:** Top ten reduced methylation peaks.

Chromosome	thickStart	thickEnd	Gene name	Fold change	Class
18	10,454,687	10,474,202	APCDD1	−9.34	CDS
4	186,708,548	186,708,998	FAT1	−8.46	CDS
9	38,397,946	38,398,155	ALDH1B1	−7.14	three_prime_utr
2	237,366,914	237,367,035	COL6A3	−6.98	CDS
15	100,900,720	100,907,186	ALDH1A3	−6.94	CDS
16	81,045,709	81,045,920	ATMIN	−6.83	three_prime_utr
16	67,828,216	67,828,456	CENPT	−6.78	exon
16	15,107,433	15,110,752	PKD1P6-NPIPP1	−6.75	exon
5	16,699,540	16,701,140	MYO10	−6.64	CDS
2	187,484,874	187,488,374	TFPI	−6.63	exon

Note: thickStart/thickEnd: start/end position of differentially methylated RNA peak; CDS, coding sequence.

### Differentially Methylated RNAs Are Involved in Important Biological Pathways

**FIGURE 2:**
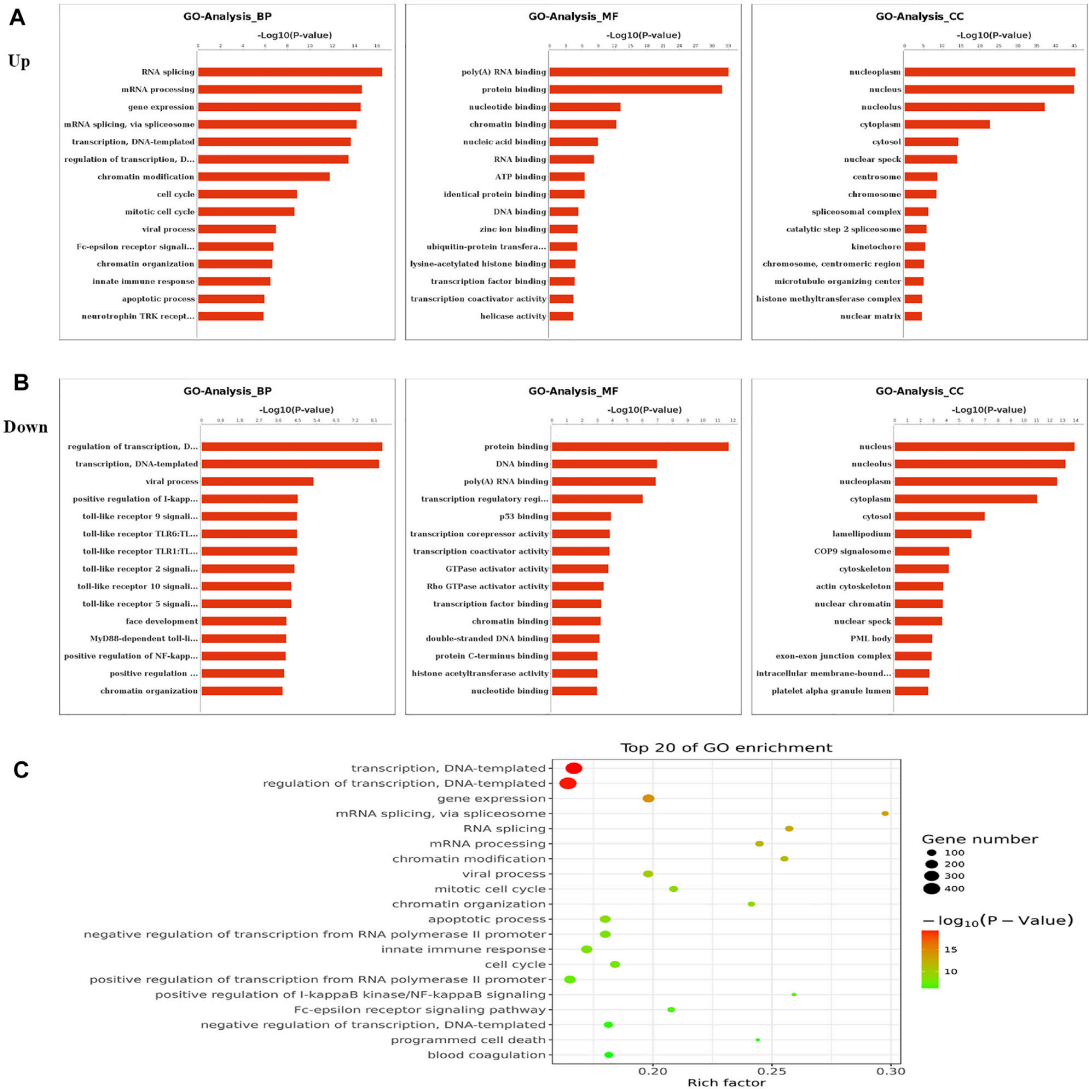
Gene ontology and Enrichment analysis of differential m^6^A peaks. **(A)** The top ten Gene Ontology terms related to mRNAs with increased methylation; terms in the biological process (BP), molecular function (MF), and cellular component (CC) categories are shown; (−log10p) for significance. The log-transformed results were used for visualization. **(B)** The top ten Gene Ontology terms related to mRNAs with decreased methylation, terms in the BP, MF and CC categories are shown; (−log10p) for significance. The log-transformed results were used for visualization. **(C)** The GO bubble shows the top 20 Gene Ontology terms corresponding to the methylated genes.

To elucidate the functions of m^6^A in NMOSD, we selected protein-coding genes containing DMMSs for GO and KEGG pathway analyses. In the BP category, genes with increased m^6^A site methylation were significantly (*p <* 0.05) enriched in RNA splicing, mRNA processing, and gene expression (Figure 2A); genes with decreased m^6^A methylation were highly enriched in the regulation of transcription, transcription, DNA templating, and viral processes (Figure 2B). In the MF category, poly(A) RNA binding and protein binding were enriched among mRNAs with increased m^6^A methylation (Figure 2A), and those with lower levels of methylation were enriched in protein binding and DNA binding (Figure 2B). With respect to the CC category, the nucleus and nucleolus showed enrichment for both increased (Figure 2A) and decreased m^6^A methylation (Figure 2B). We also used a GO bubble chart to illustrate the top twenty enriched GO terms, mainly involving transcription, DNA templating, regulation of transcription, and gene expression, among others (Figure 2C).

**FIGURE 3:**
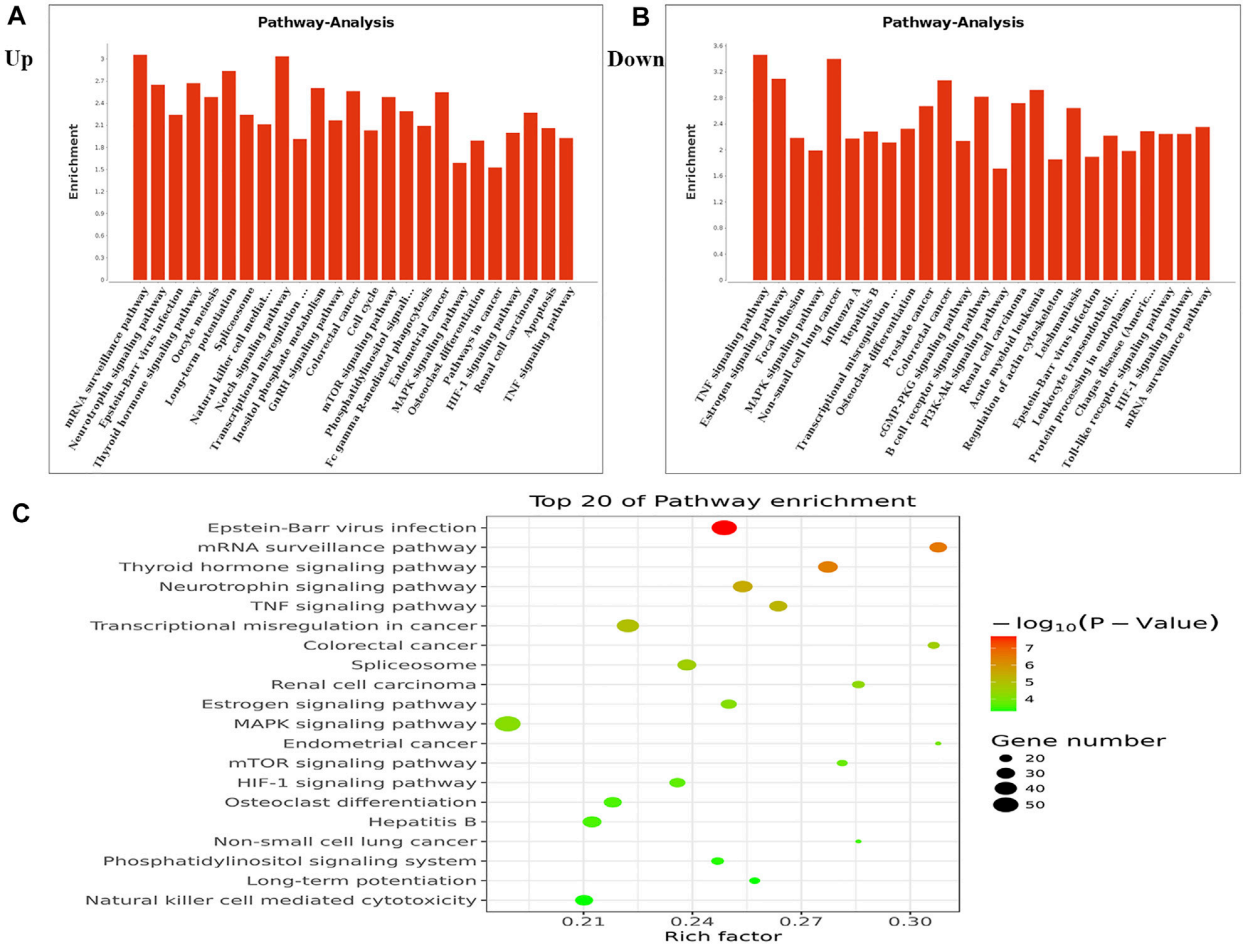
KEGG pathway and Enrichment analysis of differential m^6^A peaks. **(A)** The top ten enrichment pathway terms related to mRNAs with increased methylation are shown; (−log10p) for significance. The log-transformed results were used for visualization. **(B)** The top ten enrichment pathway terms related to mRNAs with decreased methylation; (−log10p) for significance. The log-transformed results were used for visualization. **(C)** The KEGG bubble shows the top 20 enrichment pathway terms related to methylated mRNAs.

We performed KEGG pathway analysis of DMMSs and observed that genes with increased m^6^A methylation were significantly (*p <* 0.05) enriched in the mRNA surveillance pathway, neurotrophin signalling pathway, and Epstein-Barr virus infection, among others (Figure 3A). Genes with decreased m^6^A methylation were significantly (*p <* 0.05) enriched in the TNF signalling pathway, oestrogen signalling pathway, and focal adhesion (Figure 3B). We also used a KEGG bubble chart to show the top twenty enriched pathways, including Epstein-Barr virus infection and the mRNA surveillance pathway (Figure 3C). These results suggest that m^6^A may have many key roles in NMOSD.

### Association Analysis of Differential RNA Modification and Differential Gene Expression

**FIGURE 4:**
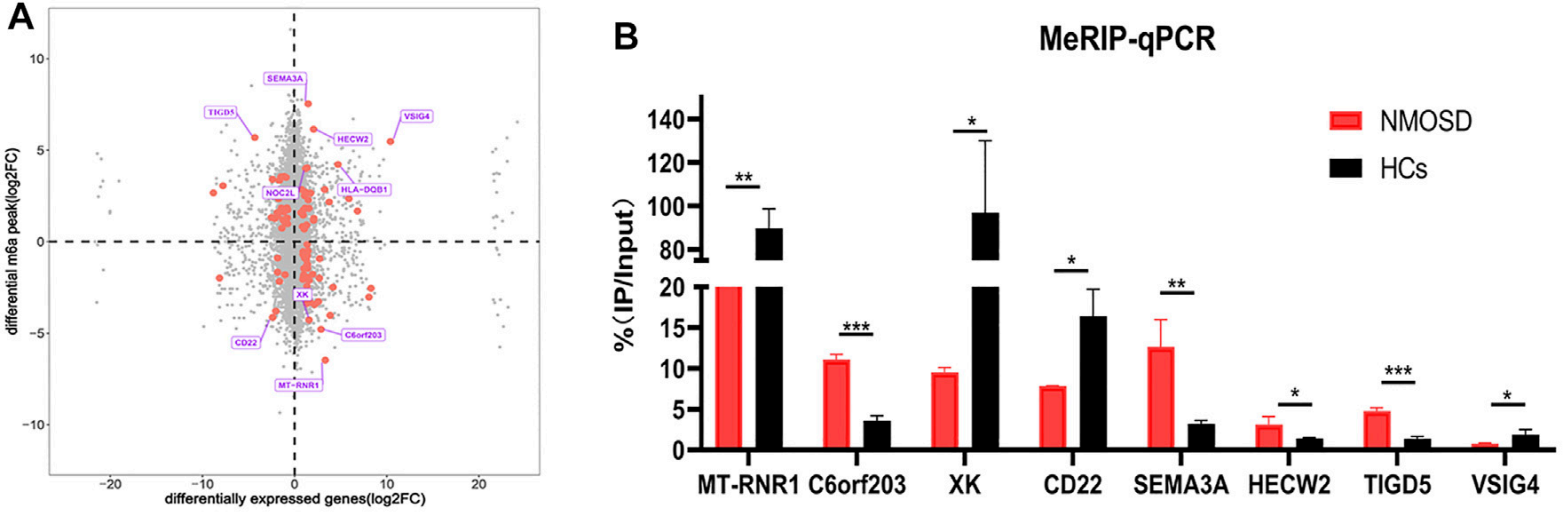
Differential RNA modification and validation. **(A)** Analysis of differential RNA modification and differential gene expression. The X-axis represents the RNA-seq differential genes, and the Y-axis represents the RNA-modified differential peak genes; the top 10 genes (largest absolute values of diff.log2. fc) are shown in purple. The red dots represent significant differences (both RNA-seq and RNA modification data meet *p* < 0.05); the grey dots indicate that the data did not meet the condition required for the red dots. **(B)** Validation of the top four hypomethylated genes and the top four hypermethylated genes by using MeRIP-qPCR.

We drew a four-quadrant map based on two sets of data: RNA-seq, all gene differential expression table (including non-significant results); and RNA modification, all differential peak table (including nonsignificant results). The top 10 genes (the largest absolute values of diff.log2. fc are shown in purple) were determined to be MT-RNR1, C60rf203, XK, CD22, SEMA3A, HECW2, TIGD5, VISIG4, HLA-DQB1 and NOC2L. (Figure 4A).

### Validation of Differential RNA Modifications by MeRIP-qPCR

In order to verify the reliability of MeRIP-seq results, MeRIP-qPCR was used to verify the modification levels of modification sites on the target genes.We chose the top four hypomethylated genes and the top four hypermethylated genes as target genes (Figure 4A). Among the eight target genes, MeRIP-qPCR results of MT-RNR1, XK, CD22, SEMA3A, HECW2 and TIGD5 were consistent with the sequencing results, and there were significant differences (*p* < 0.05) (Figure 4B). MeRIP-qPCR results of VISIG4 and C60rf203 were opposite with the sequencing results, although there were significant differences (*p* < 0.05) (Figure 4B). Nevertheless, it suggested that our sequencing results were credible.

## Discussion

m^6^A modification is a dynamic and reversible RNA modification occurring in eukaryotes that is carried out by “writers”, “erasers”, and “readers”. In recent years, studies have found that m^6^A is involved in the regulation of the immune microenvironment (Chen et al., 2019b; Tang et al., 2020; Xu et al., 2020). NMOSD is a kind of autoimmune disease that is triggered by disruption of immune homeostasis. In this study, we assessed the m^6^A status in NMOSD patients and HCs, revealing differences between the groups that support the dynamic features of m^6^A modification. Indeed, we found that the m^6^A level was much lower in patients than in HCs, demonstrating that m^6^A modification is very important in the process of NMOSD. In addition, Zhang found that altering m^6^A levels might be a novel way for improving the efficacy of platinum in cancer treatment (Zhang et al., 2021a), and Zhao reported that m^6^A regulation could influence the progression of lung adenocarcinoma (Zhao and Xie, 2021). We hypothesized that increasing the level of m^6^A might relieve the symptoms of NMOSD, but further studies are needed to confirm this hypothesis.

In the present study, many important biological pathways were found to be related to differentially methylated mRNAs. Because of the construction of strand-specific libraries, we also predicted lncRNAs harbouring m^6^A peaks and identified DMMSs. While lncRNAs are known to play important roles in mediating transcriptional and posttranscriptional regulation (Cantile et al., 2021; Moison et al., 2021), their biological functions in regards to m^6^A modification remain unknown. In general, the m^6^A modification of lncRNAs is very common in cancers. For example, Wu found that m^6^A-induced lncRNA RP11 could cause the dissemination of colorectal cancer cells (Wu et al., 2019). In our study, we investigated the m^6^A modification of lncRNAs between NMOSD patients and HCs, which highlighted immune homeostasis. However, further analysis is needed to validate these results.

**FIGURE 5:**
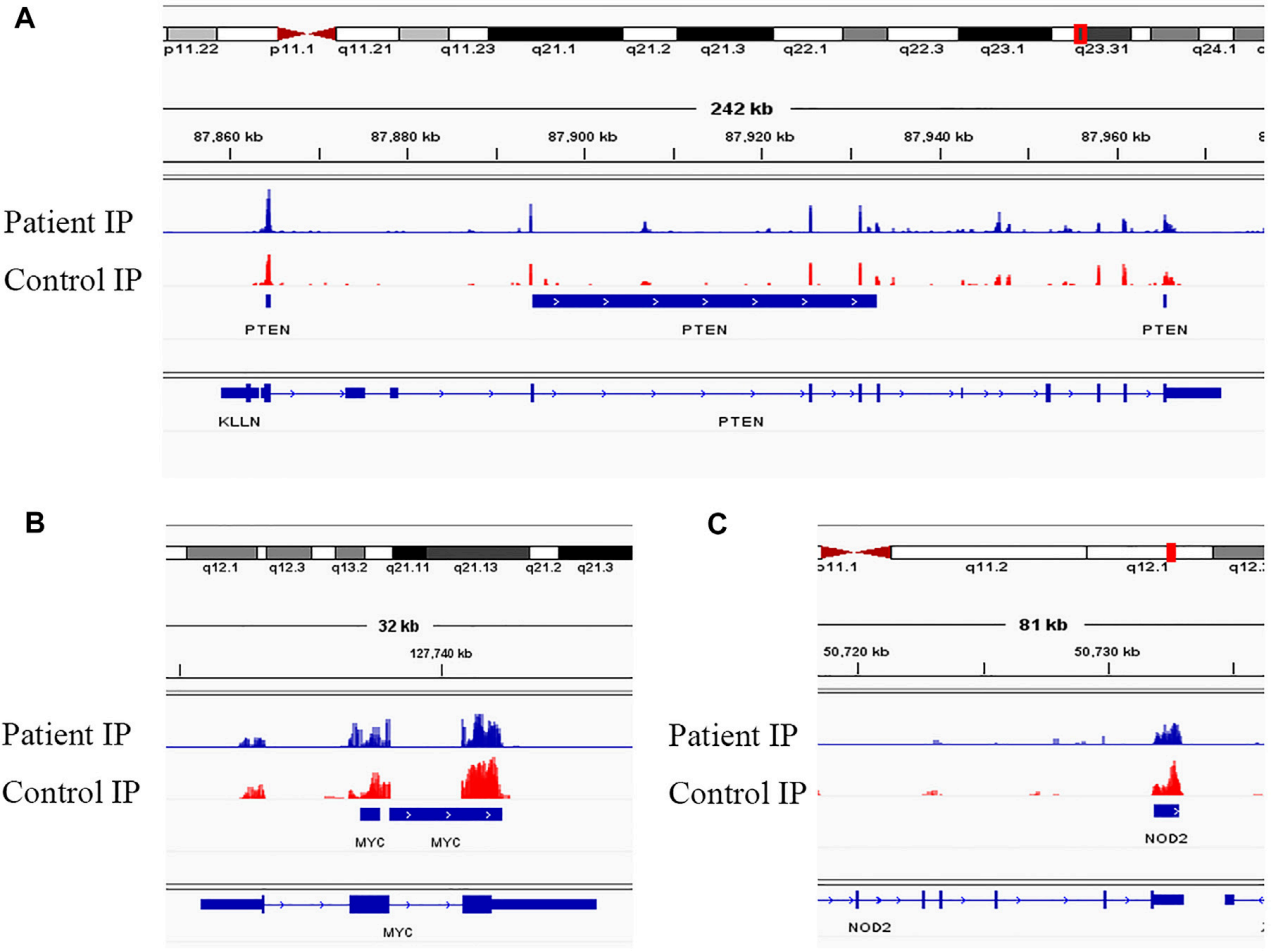
Visualization of representative genes. **(A)** Visualization of the m^6^A-modified gene PTEN in NMOSD patients and HCs. **(B)** Visualization of the m^6^A-modified gene MYC in NMOSD patients and HCs. **(C)** Visualization of the m^6^A-modified gene NOD2 in NMOSD patients and HCs.

Evidence supports a strong relationship between immune homeostasis and m^6^A modification, with m^6^A modification playing important biological roles in NMOSD (Chen et al., 2020; Chong et al., 2021; Zhang et al., 2021b; Zhong et al., 2021). In our study, the GO and KEGG analyses of mRNAs harbouring DMMSs showed that those with increased methylation were mostly enriched in RNA splicing, mRNA processing and gene expression, which supports the importance of m^6^A modification in NMOSD. For example, the increased expression of phosphatase and tensin homolog deleted on chromosome Ten (PTEN) and an approximately twofold enhancement methylation were observed in NMOSD patients compared with the HCs, indicating PTEN serves as a key enzyme in NMOSD (Figure 5A) (Han et al., 2019). Furthermore, PTEN is involved in inositol phosphate metabolism. Gene expression is important in NMOSD, and our results suggest a link between NMOSD and the regulation of gene expression, subsequently resulting the disruption of immune homeostasis.

In contrast to m^6^A sites with increased methylation, those with decreased methylation were mostly enriched in regulation of transcription, DNA templating, and positive regulation of I-kappa B kinase/NF-kappa B signalling pathways, which include genes such as v-myc avian myelocytomatosis viral oncogene homologue (MYC) (Figure 5B) and nucleotide-binding oligomerization domain containing 2 (NOD2) (Figure 5C). “Readers”, which include YTHDF1-3, recognize and bind to m^6^A modifications on mRNAs (Wang et al., 2014; Li et al., 2017; Jiang et al., 2019), thereby playing an important role in regulating translation. According to recent studies, we hypothesize that the methylation of mRNAs is associated with their translation, as it may influence their expression, leading to subsequent changes in global translation.

In our association analysis of differential RNA modification and differential gene expression, the top 10 genes included SEMA3A and CD22 (Figure 4A). Zhang also reported that SEMA3A may negatively regulate axonal regeneration in retinal ganglion cells (Zhang et al., 2020), and CD22 is an important inhibitory molecule on the surface of B cells that negatively regulates B cell activation (Gross et al., 2009). All these findings are very beneficial for future studies.

Although few studies have explored the roles of m^6^A modification in NMOSD, there are still some limitations to our study. For example, we may need to expand the number of enrolled patients. In addition, data from the same patient should be obtained before and after treatment.

## Conclusion

Our study demonstrates the differential m^6^A methylome in NMOSD patients compared to healthy controls, suggesting a strong association between m^6^A methylation and the regulation of immune homeostasis in NMOSD. The findings fundamentally contribute to future studies on immune homeostasis in NMOSD.

## Data Availability

The raw data have been made publicly available under SRA accession number PRJNA737585.
