# Supplementary material for: Comprehensive Analysis of N6-Methyladenosine (m6A) Methylation in Neuromyelitis Optica Spectrum Disorders
Source: Front Genet. 2021 Nov 11;12:735454. doi: 10.3389/fgene.2021.735454 (PMC8660110; doi:10.3389/fgene.2021.735454)
Supplement: Supplementary file 1 [file Table1.DOCX]

| Genec | Primer type | Primers |
| --- | --- | --- |
| MT-RNR1 | 1-Forward | CCACCTCTTGCTCAGCCTAT |
|  | 1-Reverse | GGCCCTGTTCAACTAAGCAC |
| C6orf203 | 2-Forward | TCTGCCGTGATCATCTCTCC |
|  | 2-Reverse | ATGATGGACCCTGGGAAGTG |
| XK | 3-Forward | GGAGGAGCTTTGAGATTGCC |
|  | 3-Reverse | CAGGACGGAGGTAAAGAGGA |
| CD22 | 4-Forward | CACACACACACTCACTGCG |
|  | 4-Reverse | GGAGTTTTGGAGGTTTGGGG |
| SEMA3A | 5-Forward | CTTCCAAAAGCAACACCATA |
|  | 5-Reverse | TCACAGATGGAAGCTCATGT |
| HECW2 | 6-Forward | ACCGTGGAGCTGATCATGTA |
|  | 6-Reverse | TGGAAGGATTTGGGTGAGCT |
| TIGD5 | 7-Forward | TGTGGTAGGAAAGGAAGGGG |
|  | 7-Reverse | CTAGGTAGGTAAGGGGTTGGG |
| VSIG4 | 8-Forward | GTGGCCAGAAACTCATAATCCA |
|  | 8-Reverse | GTACCAGATCATCGCCCAGA |

Additional file 1: Table S1. The primers used in this study
